# Imaging for Diagnosis, Monitoring, and Outcome Prediction of Large Vessel Vasculitides

**DOI:** 10.1007/s11926-020-00955-y

**Published:** 2020-09-21

**Authors:** Valentin Sebastian Schäfer, Lei Jin, Wolfgang Andreas Schmidt

**Affiliations:** 1 Department of Rheumatology and Clinical Immunology, Clinic of Internal Medicine III, University Hospital Bonn, Bonn, Germany; 3 Immanuel Krankenhaus Berlin, Medical Centre for Rheumatology Berlin-Buch, Berlin, Germany

**Keywords:** Giant cell arteritis, Takayasu arteritis, Imaging, Diagnosis, Outcome, Large vessel vasculitis, Ultrasound, Magnetic resonance imaging, Computed tomography, 18F- fluorodeoxyglucose positron emission tomography

## Abstract

**Purpose of Review:**

To discuss and summarize the latest evidence on imaging techniques in giant cell arteritis (GCA) and Takayasu arteritis (TAK). This is a report on the performance of ultrasound (US), magnetic resonance imaging (MRI), computed tomography (CT), 18F-fluorodeoxyglucose positron emission tomography (18-FDG-PET), and other emerging imaging techniques in diagnosis, outcome prediction, and monitoring of disease activity.

**Recent Findings:**

Imaging techniques have gained an important role for diagnosis of large vessel vasculitides (LVV). As signs of vasculitis, US, MRI, and CT show a homogeneous arterial wall thickening, which is mostly concentric. PET displays increased FDG uptake in inflamed artery walls. US is recommended as the initial imaging modality in GCA. MRI and PET/CT may also detect vasculitis of temporal arteries. For TAK, MRI is recommended as the first imaging modality as it provides a good overview without radiation. Extracranial LVV can be confirmed by all four modalities. In addition, MRI and PET/CT provide consistent examination of the aorta and its branches. New techniques such as contrast-enhanced ultrasound, PET/MRI, and auxiliary methods such as “computer-assisted quantitative analysis” have emerged and need to be further validated.

**Summary:**

Imaging has partly replaced histology for confirming LVV. Provided experience and adequate training, US, MRI, CT, or PET provide excellent diagnostic accuracy. Imaging results need to complement history and clinical examination. Ongoing studies are evaluating the role of imaging for monitoring and outcome measurement.

## Introduction

Giant cell arteritis (GCA) and Takayasu arteritis (TAK) are granulomatous large vessel vasculitides (LVV). They are associated with considerable morbidity. Vision loss is common in GCA if initiation of treatment is delayed. Strokes and occlusions may occur in both GCA and TAK. Early diagnosis is crucial. Compared with histology, imaging is non-invasive, and results are readily available.

Similar clinical, histopathologic, and imaging features suggest that they are somewhat related. Otherwise, GCA and TAK differ in terms of age of onset (GCA > 50 years, TAK most commonly < 40 years), sex predominance (females even more commonly affected in TAK than in GCA), distribution of arterial lesions, treatment, and prognosis. Histopathology shows granulomatous inflammation [1], and imaging of extracranial arteries often displays involvement of the aorta and its branches in both entities [2–4]. Differentiating between GCA and TAK is important, as recent studies have shown different treatment responses in GCA and TAK to the same biologic therapies [5, 6], indicating the importance of correct diagnosis. This will affect the treatment regime and subsequently the prognosis.

The age at onset is an important feature to distinguish these two diseases. TAK occurs before 40 to < 50 and GCA with > 50 years of age [3]. The temporal arteries are never affected in TAK. The axillary arteries are more commonly involved in GCA. The carotid and subclavian arteries are more commonly involved in TAK [7].

Temporal artery biopsy (TAB) has been the diagnostic gold standard for decades for confirmation of GCA, but it is invasive and lacks sensitivity with false-negative test results in up to 60%. Results may further be false-negative due to skip lesions or limitation of GCA to extracranial arteries. Furthermore, results are mostly available not earlier than 1 week after GCA has been suspected. It could be shown that the net monetary benefit was £485 per patient in favor of ultrasound (US) when compared with TAB as the first diagnostic test [8]. Obtaining a biopsy in TAK is impossible in most cases due to the predominant involvement of extracranial arteries. If surgery is necessary due to stenosis, occlusion, or aneurysms, it should be attempted in every case to harvest material from arteries for histological examination. As the diagnosis can be reliably confirmed by imaging, imaging has increasingly become the first-line diagnostic test for confirmation of GCA and TAK [2].

The role of different imaging modalities including US, magnetic resonance imaging (MRI), computed tomography (CT), and 18F-FDG positron emission tomography (18-FDG-PET) in LVV has been addressed in several studies over the last years. A European League Against Rheumatism (EULAR) project has therefore been undertaken to develop recommendations for the use of imaging in LVV in clinical practice [9].

Ultrasound-guided fast-track clinics for patients with suspected GCA have been introduced. Physicians can contact centers offering these clinics. Patients will receive an appointment within 24 h during the week. Rheumatologists experienced in GCA perform a structured history and clinical examination. This is directly followed by a US exam of at least the temporal and axillary arteries, preferably by the same rheumatologist. The incidence of vision loss significantly decreased after the introduction of such fast-track clinics [10–12]. Based on these and other considerations, the importance of imaging modalities has steadily increased [13, 14].

Ultrasound, CT, PET/CT, and MRI are increasingly applied. Older studies used angiography. Angiography however carries risks of allergic reactions, hematoma, iatrogenic embolisation, and arterial dissection, and it does not display the inflamed arterial wall itself. Therefore, modern imaging methods have almost replaced angiography unless it is performed for therapeutic vascular interventions [15]. EULAR recommendations for the use of imaging in LVV in clinical practice have been published, in which recommendations for each imaging modality were given [9].

The aim of this review is to discuss and summarize the latest evidence on different imaging modalities in diagnosis, outcome prediction, and monitoring of disease activity in both GCA and TAK.

## Methods

A literature review was conducted using PubMed, Embase, and Cochrane databases from March 2015 to March 2020. Search termini were Takayasu arteritis, giant cell arteritis, imaging, magnetic resonance imaging, computed tomography, 18F-FDG positron emission tomography, contrast-enhanced ultrasound, ultrasound, positron emission tomography/magnetic resonance imaging, large vessel vasculitis, outcome, and prognosis. Papers were included by clinical relevance.

### Imaging in Diagnosis of Giant Cell Arteritis and Takayasu Arteritis

#### Ultrasound

**Fig. 1:**
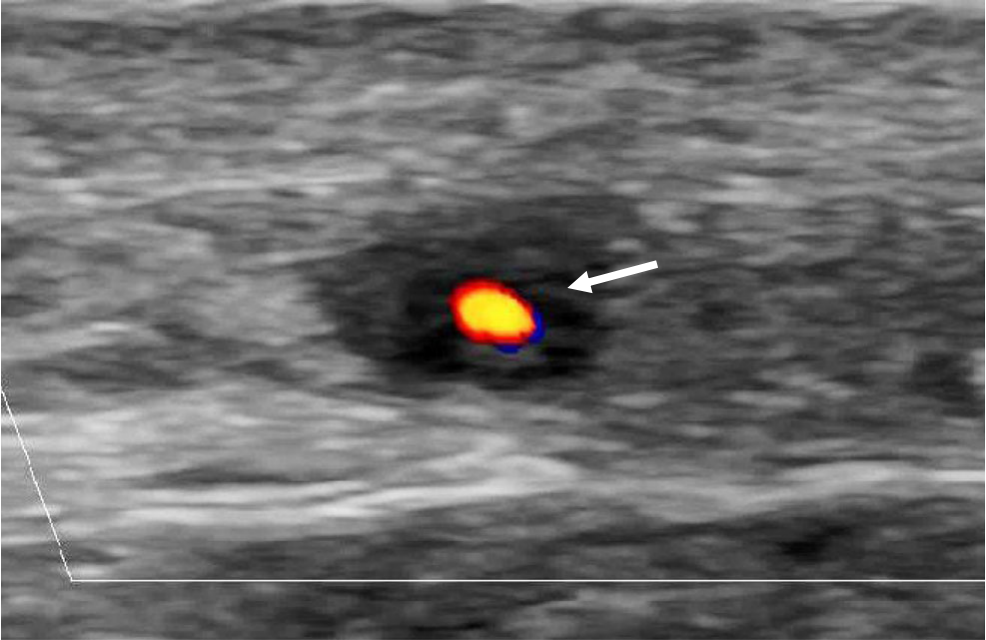
Halo sign of the common superficial temporal artery. Typical halo sign of the common superficial temporal artery, transverse scan, in a patient with newly diagnosed GCA

US can be performed simultaneously with history taking and clinical examination by the clinician and is widely used in European countries by now. Spatial resolution is very high, 0.1 mm with 20 MHz transducers and even 0.03 mm with 70 MHz transducers [16] in superficial anatomical structures such as the temporal arteries. This allows delineation of the intima-media complex (IMC) [17].. The mean intima-media thickness (IMT) in temporal and axillary arteries is 0.2 mm and 0.6 mm, respectively, in a population of 70-year-old patients. The cut-off value for distinguishing normal from vasculitic arteries is about 0.4 mm for temporal arteries and 1.0 mm for axillary arteries [18]. The “halo” and the “compression” signs are regarded as the most important US abnormalities for cranial GCA (Tables 1 and 2). The halo sign is defined as “homogenous, hypoechoic wall thickening, well delineated towards the luminal side, visible both in longitudinal and transverse planes, most commonly concentric in transverse scans” [18] (Fig. 1). The compression sign means that “the thickened arterial wall remains visible upon compression; the hypoechogenic vasculitic vessel wall thickening contrasts with the mid-echogenic to hyperechogenic surrounding tissue” [27]

**Table 1:** Definition of vasculitis in giant cell arteritis and Takayasu arteritis in different imaging modalities

Imaging modality	Giant cell arteritis	Takayasu arteritis
US	- “Halo sign” - “Compression sign” Sensitivity: 77 Sensitivity: 96 [19] (clinical diagnosis of GCA as the reference standard)	- “Macaroni sign” Sensitivity 81% Specificity of > 90% [20] (clinical diagnosis of GCA as the reference standard)
CTA	- Mural thickening and enhancement, late contrast uptake - Vascular stenosis/occlusion/ectasia - Surrounding edema/tissue reaction	Same as for GCA
	Sensitivity 84.6% Specificity 84.6% [21] (clinical diagnosis of GCA as the reference standard)	Sensitivity 100% Specificity 100% [22] (conventional angiography as the reference standard)
MRA	- Mural thickening and enhancement - Vascular stenosis/occlusion/ectasia - Surrounding edema/tissue reaction - Carotid artery involvement: branches of the external carotid artery more common [23]	Same as for GCA + Carotid artery involvement: branches of the internal carotid artery more common [23]
	Sensitivity 73% Specificity 88% [24] (clinical diagnosis of GCA as the reference standard)	Sensitivity 100% Specificity 100% [25] (conventional angiography as the reference standard)
FDG/PET	- Mural thickening and tracer uptake - Vascular stenosis/occlusion/ectasia - Surrounding edema/tissue reaction - Cluster analysis of involved arteries GCA specific: - Symmetric subclavian artery with concomitant axillary artery vasculitis	Same as for GCA + Left subclavian artery together with bilateral involvement of the carotid arteries and the mesenteric arteries [23]
	Sensitivity 92% Specificity 85% [26] (TAB as reference standard)	Sensitivity 81% Specificity 74% [20] (clinical criteria and/or angiography as the reference standard)

*US* ultrasound, *CTA* computed tomography angiography, *MRA* magnetic resonance angiography, *FDG/PET* fluorodeoxyglucose positron emission tomography

**Table 2:** Advantages and Disadvantages of different imaging modalities

Imaging modality	Advantages	Disadvantages
Ultrasound	• Widely available • Patient friendly and repeatable • Short acquisition time, approx. 15 min • Feasible for fast-track clinics • Cheaper than other imaging techniques or biopsy • Very high resolution (up to 0.1 mm in superficial anatomical structures) • High evidence level in LVV	• Limited ability to assess the thoracic and abdominal aorta • Limited overview of involved vessels
Computed tomography angiography	• Good overview of the aorta and its branches • Different contrast phases can display vasculitis • Good delineation of atherosclerotic plaques • Relatively fast acquisition time	• Radiation, approx. 17 mSv • Contract agent cannot be used in reduced kidney function
Magnetic resonance angiography	• Delineates the typical arterial pathology in LVV • Excellent overview of involved arteries • Multiple cranial and extracranial arteries can be investigated simultaneously • Superior to ultrasound for examining the aorta • Does not require ionizing radiation or iodinated contrast agents	• Less sensitive than CT and ultrasound in detecting calcifications • More expensive than ultrasound but less expensive than PET/CT • Claustrophobia, cardiac pacemakers, or other mobile-implanted metal devices • Long acquisition time • Few centers with expertise in diagnosis for cranial GCA
Positron emission tomography with computed tomography	• Excellent overview of involved arteries • Ability to detect pathology in differential diagnoses of LVV like infection or malignancy • Potentially more sensitive than MRI to detect activity in follow-up • High evidence level in LVV	• Radiation of about 25 mSv • Expensive • Not possible if glucose concentration is elevated • Sensitivity considerably decreases after > 3 days • Atherosclerosis may be misinterpreted as LVV particularly in the femoral arteries

The inter-rater and intra-rater agreement of images and videos applying the above-mentioned definitions was excellent, with mean kappa values of 0.83–0.98 for both inter-rater and intra-rater reliabilities [27]. In the TABUL study, a study to compare the diagnostic value of US with that of biopsy, readers of US images and videos obtained the same reliability as pathologists evaluating TAB specimen [8]. Further, it was shown in a live exercise on patients that these definitions are reliable in recent-onset GCA, if experienced sonographers (> 300 examinations) have 15–20 min for a standardized examination with prior training and application of > 15 MHz probes [28]. The main disadvantage of US is its limited ability to assess the thoracic aorta except for transesophageal echocardiography.

##### Ultrasound in Giant Cell Arteritis

A standardized US examination in GCA should include at least the temporal and axillary arteries. Adding the examination of axillary arteries increases the diagnostic yield of US for the diagnosis of GCA [29]. In a recently published study, the sensitivity increased from 71 to 97% when additionally examining the carotid and axillary arteries to the temporal arteries. Further arteries may be examined if the clinical diagnosis is not yet clear. Another imaging technique such as PET-CT is only needed if the diagnosis is still not confirmed or excluded after the US examination [30].

##### Ultrasound of Temporal and Axillary Arteries

**Fig. 2:**
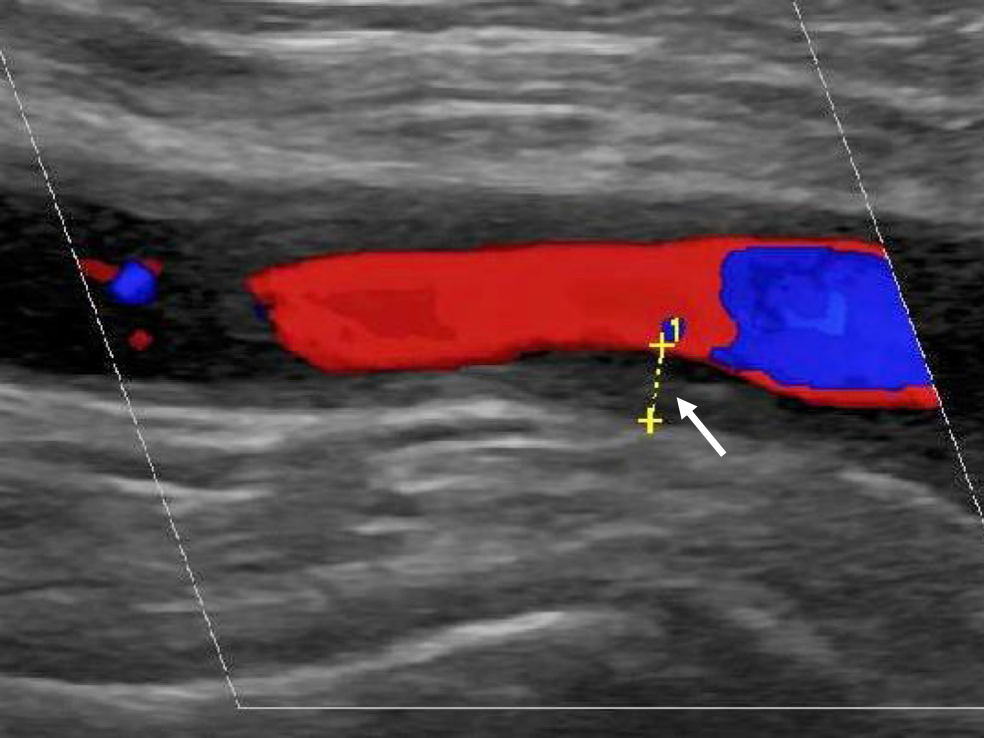
Vasculitis of the axillary artery in giant cell arteritis (longitudinal view). Longitudinal view of the axillary artery in a patient with large vessel GCA, the intima-media complex is significantly thickened, 1.81 mm; normal < 1.0 mm

US depicts a normal arterial IMC as a homogeneous, hypoechoic (dark), or anechoic (black) structure delineated by two parallel hyperechoic margins [27]. The definition of the “halo sign” [31] is given above (Figs. 1 and 2). Cut-off values for the intima-media thickness (IMT) can differentiate normal from vasculitic patients with high sensitivities and specificities [24, 32, 33]. The specific cut-off values are 0.42 mm, 0.34 mm, 0.29 mm, 0.37 mm, and 1.00 mm for the common superficial temporal arteries, the frontal and parietal branches, the facial arteries, and the axillary arteries, respectively [18].

Sundholm et al. [16] reported that they were able to distinguish the intima from the media in GCA and control subjects with a cut-off value for intima thickness of 0.06 mm when using a 55 MHz probe. They compare their findings with histology taken from the same anatomic area. Further research for very high-resolution US probes in the diagnosis of GCA is necessary.

A meta-analysis found a high specificity of 96% for US in diagnosis of GCA [24]. The halo sign of temporal arteries has been described in few patients with other diseases such as amyloidosis, atherosclerosis, and ANCA-associated vasculitides with temporal artery involvement [34]. These cases teach us the importance of history and clinical examination in connection with US in every patient with suspected GCA [35].

##### Ultrasound Findings in Other Cranial Arteries

The facial arteries are easily accessible at the level of the mandible. The occipital arteries are located posteriorly to the mastoid. Facial and occipital vasculitis has been detected by US in 41% and 31% of patients with GCA, respectively. More patients with facial arteritis had jaw claudication (71% versus 27%) and permanent blindness (24% versus 2%) compared with GCA patients without facial arteritis. Vasculitis of facial or occipital arteries usually occurred together with vasculitis of temporal arteries [36] and should be visualized, if ultrasound findings of temporal and axillary arteries are inconclusive.

##### Ultrasound Findings in Other Extracranial Arteries

The subclavian, common carotid, and vertebral arteries can be easily examined with US. In GCA, these arteries are at most affected in conjunction with temporal or axillary arteries. It is worth noting that carotid artery stenosis is rarely caused by GCA [37]. Depending on further symptoms, many other arteries like the abdominal aorta, coeliac and mesenteric arteries, and femoral and popliteal arteries are accessible by US. Aschwanden et al. [38] described vasculitic findings in 37 of 68 GCA patients in PET/CT and US, while 11 of 68 had positive findings only in US and 14 of 68 in PET/CT only. Authors concluded that PET/CT and US should be considered as complementary methods, with a second imaging modality increasing the diagnostic yield by 16–20%.

##### Ultrasound in Takayasu Arteritis

**Fig. 3:**
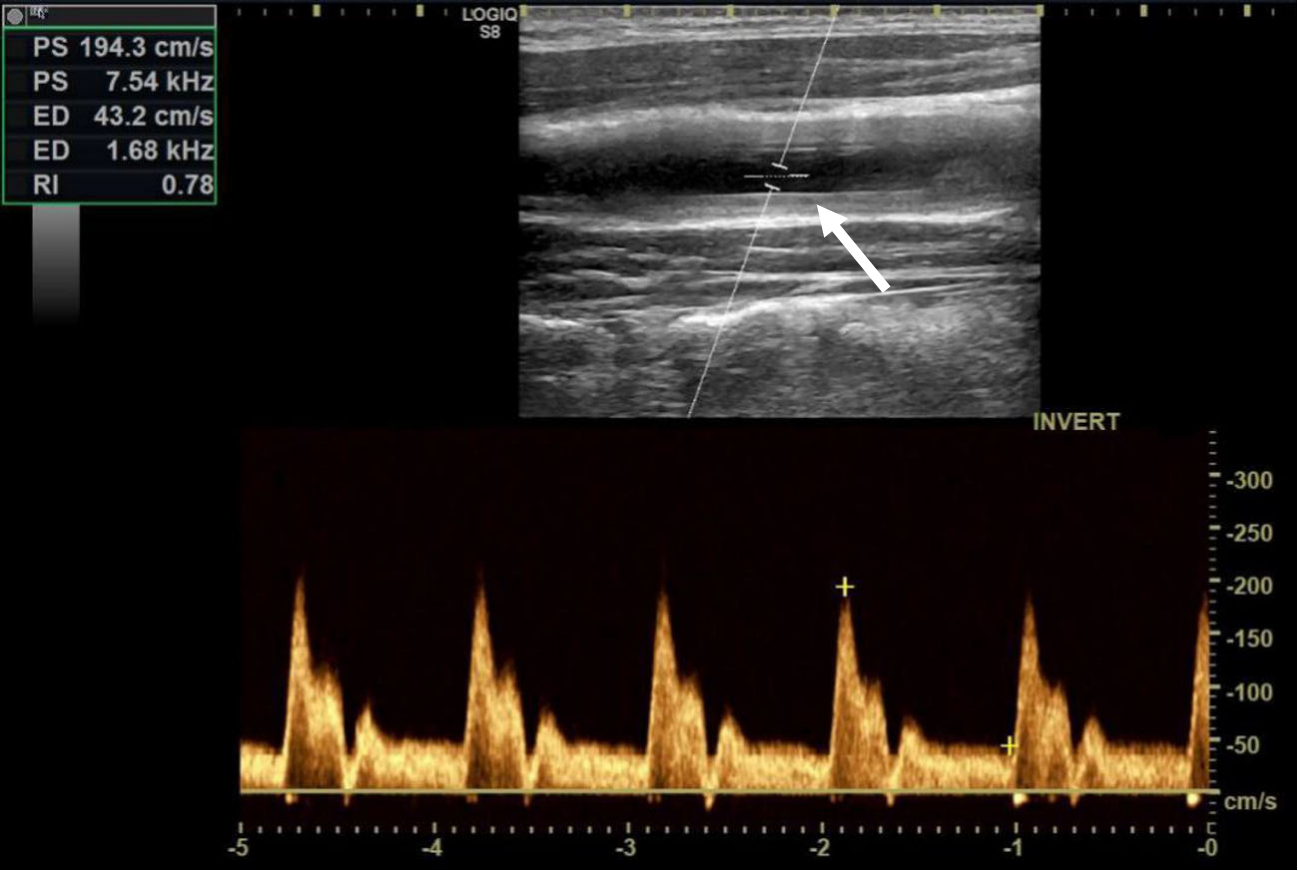
Vasculitis of the common carotid artery in Takayasu arteritis. US of the common carotid artery in a patient with Takayasu arteritis. The intima-media thickness of the common carotid artery is relevantly increased (arrow). Further is the flow velocity increased, indicating a stenosis of about 50% or decreased elasticity of the wall

In TAK, the US image is similar to GCA. The thickened artery wall appears most commonly more hyperechoic, as TAK is often diagnosed late when chronic changes are already prevalent (Fig. 3). Maeda H et al. [39]described in 1991 the “macaroni sign,” defined as characteristic circumferential arterial wall thickening of either one or both sides of the common carotid arteries, as a disease indicator for Takayasu arteritis. The “macaroni sign” is similar to the “halo sign” an indicator of an inflamed vessel wall with increased IMT.

TAK most commonly affects the left subclavian and common carotid arteries. In suspected disease, the carotid, subclavian, and vertebral arteries should be examined by US together with the abdominal aorta. In case of arterial hypertension, the renal arteries should be additionally examined. A meta-analysis on US in TAK reported a sensitivity of 81% and a specificity of > 90% for TAK diagnosis compared with clinical criteria and/or routine angiography [20].

#### Magnetic Resonance Imaging

The main advantage of MRI, which may be performed with contrast agents, is its excellent overview of involved arteries, therefore named magnetic resonance angiography (MRA). It is free from radiation. With dedicated coils, small arteries like the temporal arteries can be depicted. Its sensitivity to detect calcifications and arterioslerotic plaques is lower compared with CT and US. It cannot be performed in patients with claustrophobia, cardiac pacemakers, or other mobile-implanted metal devices or in the case of chronic kidney disease stage IV or V [40]. Further MRI is limited because of its restricted availability and its costs.

##### Magnetic Resonance Imaging in Giant Cell Arteritis

MRA provides detailed information about the arterial lumen and wall. The EULAR recommendations on imaging in LVV state that high-resolution MRI of cranial arteries may be used as an alternative for GCA diagnosis if US is not available or inconclusive. It can also be used for examining the extracranial arteries to support the diagnosis of large vessel GCA [9].

A recent meta-analysis [24] summarized 43 prospective studies (39 on GCA, 4 on TAK), six studies compared MRA with the clinical diagnosis of GCA as a reference standard; the pooled sensitivity and specificity were 73% (95% CI, 57–85%) and 88% (95% CI, 81–92%) respectively, for the detection of GCA in MRA. When TAB was used as the reference standard, MRA yielded a sensitivity of 93% (95% CI, 89 to 96) and a specificity of 81% (95% CI, 73 to 87) [24]. Data from this meta-analysis were derived only by two very specialized centers, bearing the risk of considerable bias regarding the value of this technique in general practice. In contrary to previous knowledge, vasculitis may not be limited to extracerebral arteries but may extend to intra-cerebral arteries. Intracranial arteries are, however, not solely involved in GCA [41].

Conventional two-dimensional (2D) black-blood sequences are time-consuming, provide a limited scan area, and cannot be reconstructed in various planes. Thus, vessels which are oriented obliquely cannot be analyzed perpendicularly to their course. Recently, a high-resolution T1w three-dimensional (T1w-3D) fat-suppressed turbo spin echo (TSE) sequence (VISTA volumetric isotropic TSE acquisition) has resolved these limitations. Treitl et al. [42] examined 25 LVV patients with a 3 T MRA using 1.2 × 1.3 × 2.0 mm^3^ fat-suppressed, T1w-3D, modified volumetric isotropic TSE acquisition (mVISTA) pre- and post-contrast sequences. Authors concluded that navigated fat-suppressed T1w-3D black-blood MRI with PPU-triggering allows diagnosis of thoracic LVV with good reliability results. Unfortunately this technique is not available in routine clinical practice.

##### Magnetic Resonance Imaging in Takayasu Arteritis

The EULAR recommendations propose MRA as the first imaging modality in diagnosis of TAK [9]. This recommendation is almost entirely based on expert opinion and current clinical practice. MRI is a technique without radiation exposure and therefore preferable over other imaging modalities in the rather young TAK patients. MRI enables the assessment of the vessel wall and luminal changes, which are both relevant for TAK, and provides an information on the distribution of vessel involvement. One study comparing MRA with angiography as the reference standard yielded a sensitivity of 98% and a specificity of 100% for MRA in TAK [25].

#### 18-Fluorodeoxyglucose Positron Emission Tomography

18-FDG-PET detects increased glucose metabolism in inflamed arteries. It is most commonly combined with CT in order to allocate PET findings to a specific arterial segment. PET-CT provides an excellent overview in suspected vasculitis. Particularly in patients with unclear inflammation, PET may detect alternative diagnoses like tumors, lymphoma, or septic foci [43]. PET should be performed not later than 3 days after initiation of glucocorticoid treatment as sensitivity considerably decreases. In patients with initially positive PET-CT, only 36% had positive findings after 10 days [20]. PET-CT is however expensive, and radiation exposition is high. Its use is limited with elevated blood glucose levels.

##### 18-FDG Positron Emission Tomography in Giant Cell Arteritis

Until recently, PET was thought to be limited to extracranial arteries. New studies however showed that modern equipment can detect pathology in smaller arteries like temporal, facial, and maxillary arteries [26, 44].

In large vessel GCA, the subclavian arteries display most frequently FDG uptake (up to 75%), followed by the abdominal and thoracic aorta in approximately 50%, while an increased FDG uptake in the axillary, carotid, iliac, and femoral arteries is seen in 30–40% [45]. One study arrived at a specificity of > 90% in the supraaortic arteries but lower specificities for the aorta and the lower extremities (70–80%). FDG uptake is typically symmetrical in GCA. The specificity of increased FDG uptake in the lower limbs and the abdominal aorta is lower (70–80%), because these arteries are more prone to atherosclerosis. As glucose metabolism is increased in the arterial walls in arteriosclerosis, PET can be falsely positive, albeit enhancement is usually less intense in arteriosclerosis.

In a recent study of 64 newly suspected GCA patients, the sensitivity and specificity of PET/CT were 92% and 85%, respectively, compared with TAB, with a high negative predictive value [26, 46]. A recent retrospective study evaluated the diagnostic performance of 18-FDG PET-CT for large vessel involvement in patients with suspected GCA and negative TAB [47]. In these 63 patients, 18F-FDG PET-CT showed large vessel involvement in 22 patients, 14 of whom were finally diagnosed with GCA and 41 patients were 18F-FDG PET-CT negative and nine were diagnosed with GCA.

Although the intensity of vascular FDG uptake in GCA declines with glucocorticoid treatment, long-term persistent vascular FDG uptake may be present despite clinical remission [48]. Blockmans D et al. [45] suggested that FDG-PET cannot identify patients at risk of relapse. Arnaud et al. [49] reported a poor correlation between FDG uptake and disease activity in LVV. There are arguments for ongoing subclinical inflammation, but the increased signal may also be ascribed to vascular remodeling as vascular smooth muscle cells also take up FDG [50]. Grayson et al. [51] found a residual FDG uptake in 55% of patients in clinical remission.

**Fig. 4:**
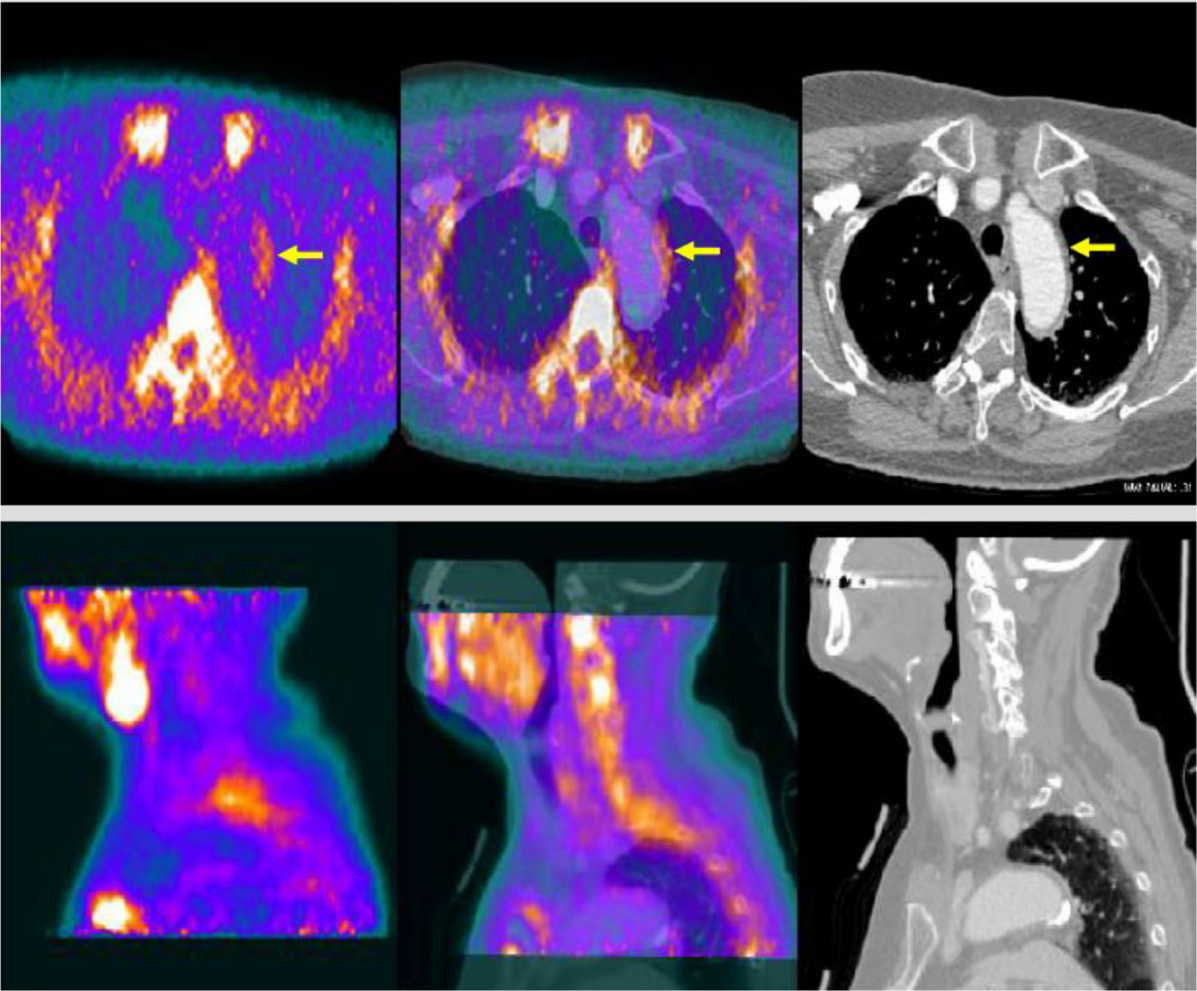
Hybrid [11C]-PK11195 PET/CT in a patient with giant cell arteritis. Hybrid [11C]-PK11195 PET-CT and CT images. From left to right, fusion image, in the middle is PK11195 and on the right side CT images of the same giant cell arteritis patient in transverse and sagittal section. Courtesy of Professor Paolo G. Camici, Milano, Italy

Activated macrophages and T lymphocytes are fundamental elements in the pathogenesis of GCA and TAK. Recently a new compound PK11195, which has a high affinity with cells of an activated nuclear phagocytic cell line, was tested and was able to visualize vascular inflammation in patients with vasculitis vs. controls. The results were not compared with conventional PET/CT due to radiation (Fig. 4) [52].

##### 18-FDG Positron Emission Tomography in Takayasu Arteritis

Incerti E et al. [53] prospectively examined 30 patients with TAK by both PET/CT and MRI. The authors concluded that PET/CT reveals unique and fundamental features of arterial involvement and confirmed the role of PET/CT in the assessment of local inflammatory and vascular remodeling during follow-up, even in lesions in which the arterial wall is < 4 mm in MRI. Soussan M et al. [54] performed a meta-analysis of studies published between January 2000 to December 2013 (21 studies, 413 patients, 299 controls) and retained a sensitivity and specificity of 87% and 73%, respectively, for discriminating active from inactive TAK.

In a recently published meta-analysis on imaging modalities for the diagnosis and disease activity assessment of TAK, pooled sensitivity of FDG-PET for disease activity was 81% (95% CI, 69–89%) and pooled specificity 74% (95% CI, 55–86%) [20]. Heterogeneity of PET/CT results could be explained by varying definitions of abnormal thresholds, patient characteristics, and standards for determining disease activity, as there is no gold standard for disease activity.

#### Computed Tomography Angiography in Giant Cell Arteritis and Takayasu Arteritis

Computed tomography angiography (CTA), requiring IV application of iodine-based contrast agents, is also a common method used in diagnosis of LVV. Arteritis on CTA presents with mural thickening and double ring enhancement after intravenous injection of iodine-based contrast agent [37]. In a prospective study on 24 patients with suspected GCA, of whom 15 were ultimately diagnosed as GCA on an individual basis by experienced clinicians, mural thickening on CTA had a somewhat lower specificity (84.6% versus 100%) and a positive predictive value of (84.6% versus 100%) than an increased FDG uptake on PET scanning, whereas sensitivity reached 73.3% for CTA and 66.7% for FDG-PET [21]. De Boysson et al. [55] compared CTA with FDG-PET/CT in a series of 28 patients with GCA. Using FDG-PET/CT as a reference, CTA showed excellent sensitivity (95%) and specificity (100%) in a per-patient analysis. In a per-segment analysis, sensitivity and specificity were 61% and 97.9%, respectively. Hommada et al. [56] described a perfect agreement between PET and CT at a patient-based level and very good agreement at a vascular segment-based level (kappa, 0.72 to 1). Discrepancies between PET and CT were observed only in relapsing GCA (*n* = 3).

## Other Methods

### Contrast-Enhanced Ultrasound

Although MRI and CT can reveal signs suggestive of vasculitis, no clear correlation with disease activity or progression has been found [13], while utility of PET in the follow-up of patients with LVV is somewhat more debated.

There is a need to develop alternative imaging modalities to assess the arterial inflammation in LVV. Therefore, some authors performed studies on contrast-enhanced ultrasound (CEUS).

In the study of Germanò G et al. [57], 31 patients (14 with TAK,17 with CCA) underwent both PET, color Doppler US, and CEUS of the right carotid artery. 18F-FDG uptake was used as the reference standard for vascular inflammation. Carotid CEUS had a sensitivity of 100% (95%confidence interval (95% CI), 65–100) and a specificity of 92% (95% CI, 72–99). Ling-Ying Ma et al. [58] compared acute phase reactants and CEUS scans of 84 TAK patients at baseline and after 3 months of therapy. They showed that the combination of CEUS parameters and ESR could help to differentiate between active and inactive TAK by physicians global assessment with a sensitivity and specificity of 81.1% and 81.5%, respectively [59]. In a study by Lottspeich et al. [60], the carotid CEUS scores decreased sharply in three patients with TAK after tocilizumab treatment. Therefore, CEUS appears to have some potential for assessing disease activity in TAK and during follow-up.

CEUS is able to investigate a limited number of vessels only, due to the short time interval when contrast agent remains at sufficient high concentration in the circulation. Most commonly, the carotid arteries have been examined. The analysis is qualitative, and results may rely on the sonographer’s experience. To overcome this deficiency, Hu Yanlu et al. [61] published a computer-assisted quantitative analysis of the carotid artery in TAK based on CEUS. First, the vasculitis lesion was outlined on the carotid wall, and one homogeneous rectangle and one polygon were selected as two reference regions in the carotid lumen. The temporal and spatial features of the lesion region and the reference regions were then calculated. Furthermore, the difference and ratio of the features between the lesion and the reference regions were computed as new features (to eliminate interference factors). Finally, the correlation was analyzed between the CEUS features and inflammation biomarkers consisting of erythrocyte sedimentation rate (ESR) and C-reactive protein (CRP). Further studies are needed to evaluate this new method.

### Positron Emission Tomography with Magnetic Resonance Imaging

Compared with PET-CT (radiation exposure in average approx. 25 mSv) [62], PET/MRI can reduce the radiation dose for patients by approximately 20 mSv, allowing comprehensive and multimodal analysis of vascular wall inflammation and vascular lumen. It offers promising perspectives for evaluating the disease activity during follow-up.

**Fig. 5:**
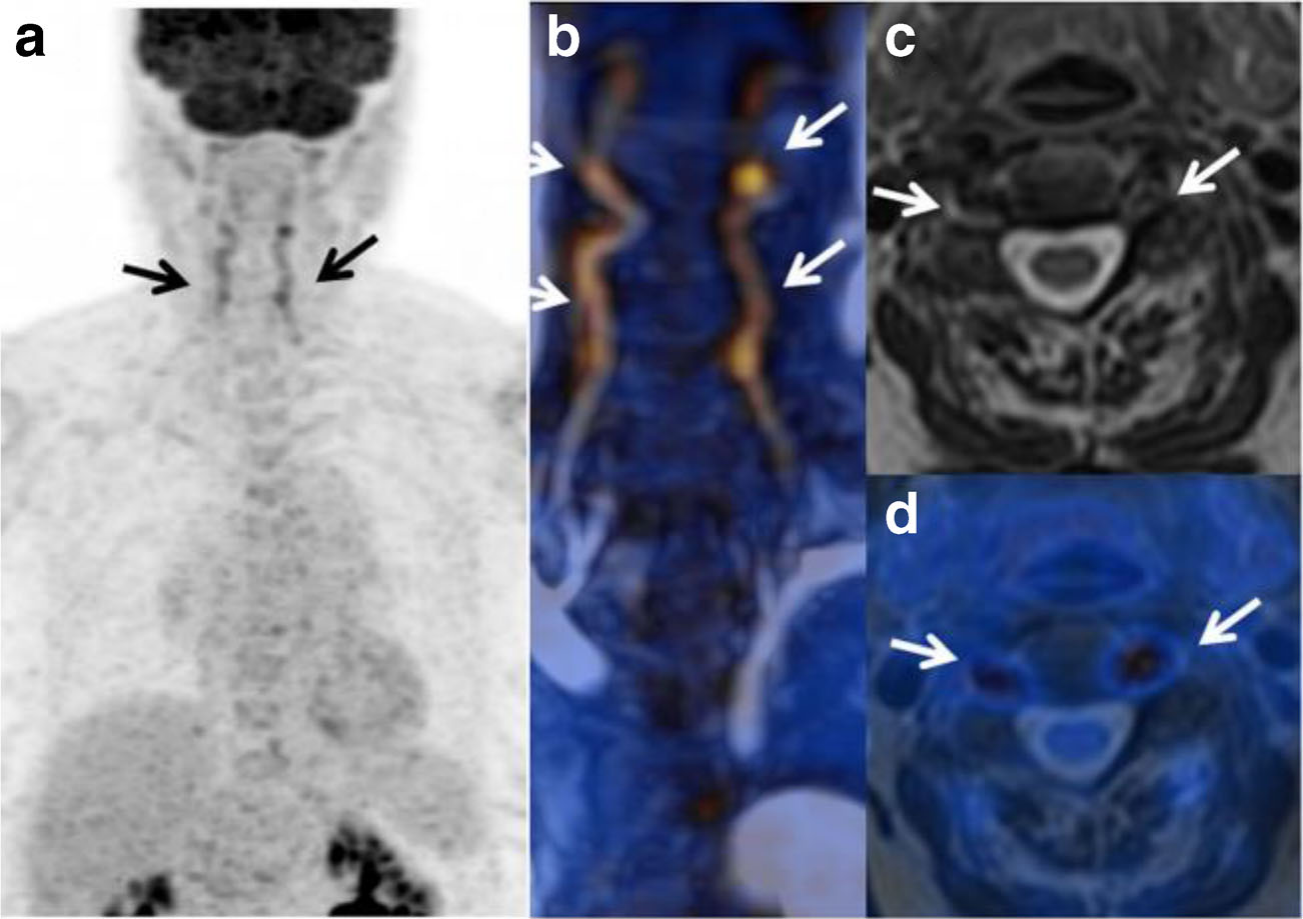
PET/MRI in a patient with giant cell arteritis. PET/MRI shows an inflammatory pattern with clear uptake (> liver uptake, grade 3) in vertebral arteries. **a** Maximum intensity projection and **b** fusion MR angiography/PET (arrows) associated with arterial wall thickening on: (**c**) MR axial T2-weighted image and (**d**) T2-weighted/PET fusion. Arrows indicate vertebral artery. Laurent C et al. Sci Rep. 2019 Aug 27;9(1):12388 [63] published under a CC BY 4.0 license

**Fig. 6:**
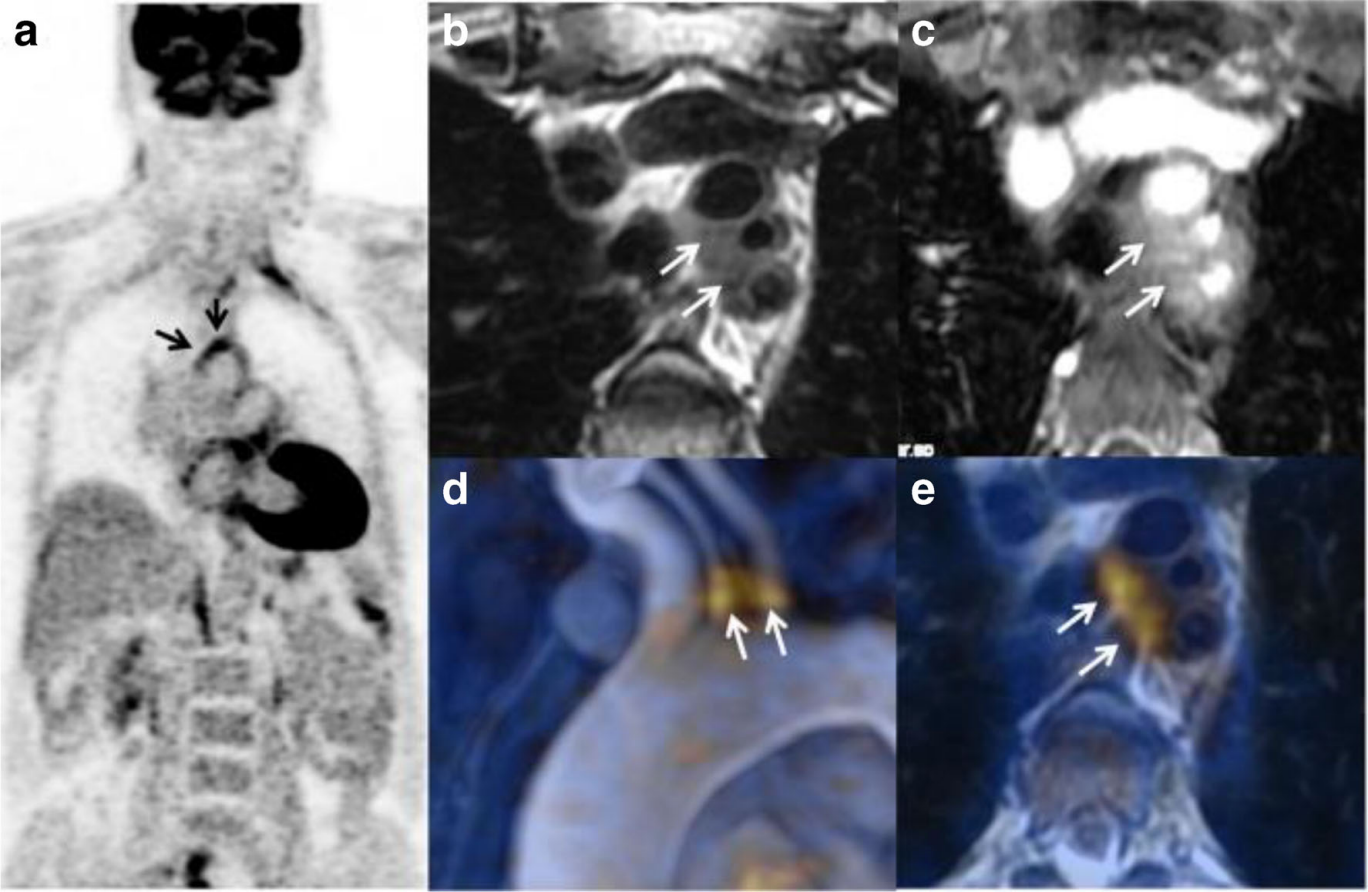
PET / MRI in a patient with Takayasu arteritis. PET/MRI ((**a**) coronal PET, (**b**) T2-weighted image, (**c**) post-contrast T1-weighted image, (**d**) fusion MR angiography/PET, (**e**) fusion PET/T2-weighted image) shows an inflammatory pattern with clear uptake (grade 3) at the origin of supraaortic arteries associated with arterial wall thickening on T2-weighted image (**a**, arrows) and wall enhancement (**b**, arrows). Fusion images (**c**, **d**) show excellent co-registration of FDG uptake and MR findings. Laurent C et al. Sci Rep. 2019 Aug 27;9(1):12388 [63] published under a CC BY 4.0 license

Both for GCA and TAK, repeated PET/MRI might help to identify relapse, progression of damage, and development of aneurysms. Additionally, MRI allows the analysis of vascular arterial gadolinium uptake as an additional marker of vascular inflammation (Figs. 5 and 6) [63].

Einspieler I et al. [64] performed PET/MRI and PET/CT in 12 LVV patients, 2 with TAK, and 10 with GCA. They compared the visual scores and quantitative parameters (maximum standardized uptake value (SUVmax) and target to background ratio) between the two methods. Authors did not find a significant difference between both modalities concerning these parameters.

In a recent retrospective study, 14 patients with aortitis (11 active GCA, 3 acitive TAK) and 14 patients with suspected active LVV underwent 18F-FDG for the evaluation of inflammatory aortic involvement. All patients were imaged with a 3 T MRI with T1W VIBE pre- and post-contrast sequences in order to compare these two imaging techniques. T1W VIBE MRI of the aorta detected vessel wall inflammation in a comparable number of patients with LVV compared with 18F-FDG PET [65]. In the retrospective study of Laurent et al. [63], 13 patients who underwent 18 PET/MRI scans (TAK, *n* = 10 scans; GCA, *n* = 8 scans) at diagnosis (*n* = 4), relapse (*n* = 7), or during remission (*n* = 7), compared PET/MRI imaging with clinical symptoms and outcome, concluded that PET/MRI was highly linked to disease activity, particularly in TAK.

## Outcome Prediction and Monitoring of Disease Activity and Damage

The lack of standardized outcome measures is problematic in the design and interpretation of clinical trials. Generic instruments like the Birmingham Vasculitis Activity Score have limitations in GCA [66], while the Indian ITAS may be helpful in TAK [67]. An international collaborative effort is underway to address this [68]. EULAR recommendations have been published for a core data set to support observational research and clinical care in GCA [69]. In a Delphi survey of international experts in LVV from different specialties, 22% stated that GCA and TAK were unsuitable for common outcome parameters [70]. The role of imaging in monitoring of disease activity in GCA and TAK is still a matter of further research.

### Ultrasound

The role of US in monitoring disease activity is currently studied. With treatment, the echogenicity of the artery walls increases, while IMT decreases. While US abnormalities normalize rapidly in temporal arteries, residual wall swelling in extracranial arteries remains visible in the majority of patients for months and years [71–73].

In large vessel GCA, IMT may be measured twice yearly [17]. If the treatment is effective, vasculitic wall thickening will become brighter and IMT decreases [74]. Low echogenicity and increasing wall thickness or new stenoses may indicate active disease. There are reports documenting resolution of the halo sign in the temporal arteries only 2 days after starting glucocorticoids; however, also a persistence of the halo sign for more than 6 months after treatment initiation has been observed [72]. In the temporal artery biopsy versus ultrasound in diagnosis of GCA (TABUL) study, a cross-sectional analysis was performed on GCA patients with a halo sign in at least one branch of the temporal artery (*n* = 131) [14]. The halo size was smaller in patients who had already been treated with glucocorticoids for up to 7 days compared with patients without treatment of a very short time of treatment before the ultrasound examination [14, 72].

In TAK, serial measurements of the common carotid artery IMT have been proposed to assess response to treatment. Active lesions were found to have a mean IMT of 3.3 ± 0.8 mm and inactive lesions of 1.6 ± 0.4 mm; in addition, increase of wall echogenicity has also been associated with decreased inflammation in TAK [72].

### Magnetic Resonance Imaging

A retrospective study by Spira D et al. [75] suggested that contrast-enhanced MRA may be useful for monitoring disease activity in primary LVV with biological therapies, as the wall thickness significantly decreased at follow-up. Yet another study showed that MRI reveals vessel wall edema also in patients who were considered to be in clinical remission, suggesting that edema does not correlate with disease activity [20]. A more recent study assessing 20 patients with TAK prospectively found a statistically significant correlation between MRI features, including wall thickening and enhancement, suggesting active disease, as well as the Indian Takayasu activity score (ITAS) [76]. Another study found no statistically significant difference in MRI parameters in 30 patients with active TAK and 19 patients with inactive TAK [77].

A numerical damage index was developed and named as the Combined Arteritis Damage Score (CARDS). This index was derived from the following formula for 25 arterial regions: number of regions with mild stenosis × 0.6 + number of regions with moderate to severe stenosis × 1.2 + number of regions with occlusions × 1.6 + number of regions with aneurysms × 0.8 [78], which might help in future trials to standardize follow-up, at the moment lacking validation.

A recent study using delayed contrast-enhanced (DCE) MRI examined 27 patients with clinically active TAK compared with 12 patients with clinically inactive TAK and 27 age- and sex-matched healthy controls [79]. Neither stenosis nor delayed enhancement of arterial wall was shown in the control group. On MRI, delayed enhancement of arterial walls could be observed in the active TA group but not in the stable TA group or the control group. Authors therefore suggested that delayed enhancement on DCE-MRI is one characteristic of the active TA.

Commonly gadolinium-based contrast agents are used for MRA. Gadofosveset is a different contrast agent that might better differentiate between active and chronic vasculitis, as it does not enhance the fibrous tissue [80]. Further studies are needed to confirm the findings of this hypothesis.

### 18-Fluorodeoxyglucose Positron Emission Tomography

18FDG PET-CT is highly sensitive for the diagnosis of GCA and TAK, but due to radiation exposition, its application for follow-up in GCA and particularly in younger patients with TAK is limited. The data regarding whether FDG-PET can be used reliably to monitor treatment response and disease activity in LVV are less certain [81]. In a retrospective study [82], seven patients with an initial positive FDG-PET scan for LVV received repeated imaging to monitor treatment response. Four out of seven patients showed no FDG uptake on subsequent scans after an initial course of prednisolone therapy, suggesting that the use of FDG-PET in monitoring disease activity and treatment response may be appropriate in assisting prednisolone dose titration. Another study suggested that imaging acquisition time significantly influences reader interpretation of disease activity in PET scans performed in patients with LVV [83]; delayed imaging allows time for FDG distribution into the arterial wall with simultaneous elimination from blood pool [84]. Recent guidelines for FDG-PET assess at least 60 min and preferably 90 min [9, 85]. The role of 18F-FDG PET/CT for monitoring disease activity and guide treatment strategies is yet to be determined. Even though arterial FDG uptake rapidly decreases under glucocorticoid treatment, 18F-FDG PET/CT performed during the disease course shows persistent pathological arterial FDG uptake in the majority of patients, even in patients considered otherwise in clinical remission [48, 51, 86]. Remodeling or smoldering inflammation is thought to be possible explanations for this arterial metabolic activity. Serial PET scans during the disease course have reported a higher incidence of subsequent relapse among patients with high composite arterial PET scores (PETVAS) [48]. Also, PETVAS scores are inversely associated to preceding treatment changes [87].These findings support the hypothesis that persistent FDG uptake may reflect smoldering inflammatory activity, but data are still too scarce to establish specific criteria to guide treatment decisions.

### Computed Tomography/Computed Tomography Angiography

Although widely used in the assessment of patients with TAK, there have been no reports comparing CTA with detailed assessment of disease activity in TAK. Sergio Prieto-González et al. [88] prospectively evaluated the outcome of CTA signs of large vessel inflammation and remodeling in GCA patients after approximately 1 year of glucocorticoid treatment. While contrast enhancement resolved in the majority of patients, vessel wall thickening persisted in two thirds. However, the number of affected aortic segments as well as aortic wall thickness significantly decreased.

## Conclusion

With the development of more sophisticated imaging technology, clinicians are provided with more and more imaging data. How to balance the advantages and disadvantages and master the indications of different imaging methods has become a must for rheumatologists.

As sign of vasculitis, US, MRI, and CT show a homogeneous arterial wall thickening, which is most commonly concentric. PET shows increased FDG uptake in inflammatory artery walls. US is recommended as the initial imaging modality in GCA, while MRI is recommended as the initial imaging modality in TAK; extracranial disease can be confirmed by all four modalities. In addition, MRI and PET/CT provide consistent and synchronized examination of the aorta and its branches. Imaging techniques have already gained an important role in the diagnosis LVV, while its role in monitoring has to be assessed in future trials. CEUS, PET/MRI, and other auxiliary methods such as “computer-assisted quantitative analysis” have emerged in the diagnosis and detection of diseases, giving a foundation for future research.

More prospective data on imaging techniques in GCA and TAK are needed, as well as on the role of imaging for outcome prediction and monitoring in LVV.
